# Targeting lysine methyltransferase 2C deficiency: New frontiers in breast cancer therapy and prognosis

**DOI:** 10.1002/ctm2.70617

**Published:** 2026-02-11

**Authors:** Shuangmei Tong, Qiu Wang, Yun Chen, Zunjie Bo, Ya Zhang, Yan Liu

**Affiliations:** ^1^ Department of Clinical Pharmacy Shanghai General Hospital Shanghai Jiao Tong University School of Medicine Shanghai China; ^2^ College of Pharmacy Dalian Medical University Dalian China

**Keywords:** breast cancer, KMT2C, therapy

## Abstract

Loss‐of‐function mutations in lysine methyltransferase 2 (KMT2C) are frequently observed in breast cancer and are significantly associated with enhanced tumour heterogeneity, aggressive clinical behaviour, and poor prognosis. Although KMT2C has been established as a tumour suppressor, the precise molecular mechanisms by which its deficiency drives metastasis and therapy resistance remain incompletely understood. This review comprehensively examines the multifaceted roles of KMT2C in breast cancer pathogenesis, with a particular emphasis on its interplay with critical oncogenic signalling networks and functional axes, including the IFNγ‐EMT/MET axis, the MAPK/ERK and RAS/PI3K/AKT/mTOR pathways, and the KDM6A–MMP3 regulatory circuit. Furthermore, we discuss emerging therapeutic strategies for KMT2C‐deficient breast cancers, such as epigenetic modulators (EZH2 inhibitors, KDM6A inhibitors, and BET inhibitors), DNA damage response (DDR)‐targeting agents, and pathway‐specific inhibitors. These insights not only elucidate the complex biological functions of KMT2C in breast cancer but also provide a rationale for developing precision therapies tailored to KMT2C‐mutant tumours.

## Graphical abstract

This graphical abstract illustrates the role of KMT2C loss‐of‐function in promoting breast cancer progression and therapeutic vulnerability. KMT2C deficiency drives tumor heterogeneity, metastasis, and therapy resistance by activating oncogenic pathways such as IFNγEMT/MET and MAPK/ERKRAS/PI3K/AKT/mTOR, along with epigenetic remodeling via the KDM6AMMP3 axis. These alterations concurrently create opportunities for targeted treatment using epigenetic drugs, DDR inhibitors, and pathway‐specific therapies.

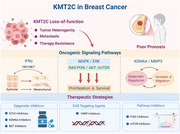

## 1 BACKGROUND

Breast cancer is the most prevalent malignancy in women and a major contributor to cancer‐related mortality.^1^ In 2022, the global age‐standardised incidence rate (ASIR) of breast cancer was 46. 8 per 100 000 population, and the age‐standardised mortality rate (ASMR) was 12. 7 per 100 000.^2^ Metastasis is the main cause of death in breast cancer.^3^, ^4^, ^5^ As a highly heterogeneous disease, breast cancer presents with diverse clinical features including breast masses, skin changes, nipple discharge, or localised pain. This heterogeneity is reflected in its molecular classification into distinct subtypes: hormone receptor‐positive/HER2‐negative (HR+/HER2–), HER2‐positive (HER2+), and triple‐negative breast cancer (TNBC). These subtypes exhibit distinct clinical features and metastatic patterns: the HR+/HER2– subtype generally has a more favourable prognosis with later‐onset metastasis, frequently to bone; the HER2+ subtype, while aggressive and prone to brain metastasis, has seen significantly improved outcomes with anti‐HER2 targeted therapies like trastuzumab, albeit with some patients developing resistance^6^; and the TNBC subtype, lacking effective targeted therapies, is the most aggressive, often leading to multi‐organ metastasis and the poorest prognosis.^7^, ^8^ Corresponding treatment strategies encompass surgery (breast‐conserving or mastectomy), chemotherapy, radiotherapy, endocrine therapy (aromatase inhibitors), targeted therapy (anti‐HER2 agents), and immunotherapy.^9^ This clinical diversity emerges from an intricate molecular pathogenesis characterised by coordinated deregulation of genomic and epigenomic programs. These intertwined genetic and epigenetic abnormalities disrupt transcriptional programs, activate pro‐tumourigenic signalling pathways, and collectively promote tumour heterogeneity, malignant progression, and therapeutic resistance.^10^, ^11^

Among the key epigenetic regulators frequently altered in breast cancer, the histone lysine methyltransferase KMT2C represents a prominent example. KMT2C undergoes loss‐of‐function mutations in approximately 12% of breast cancer cases,^12^ and aberrant expression of KMT2C may contribute to gene regulation by altering histone modifications, which in turn affects enhancer activity, thereby affecting breast cancer prognosis, promoting distant metastasis,^13^ and modulating sensitivity to chemotherapy.^14^ Unlike its homolog KMT2D, the absence of KMT2C is not only a driving factor for lung metastasis in prostate cancer,^15^ but also its expression is negatively correlated with the metastatic potential in breast cancer cell lines (*p *= .0041). In contrast, KMT2D shows no significant association with metastasis (*p *= .1857),^13^ highlighting a unique tumour‐suppressive role for KMT2C in cancer progression.

## 2 STRUCTURE AND MOLECULAR FUNCTION OF KMT2C

### 2.1 KMT2C gene and protein structure

**FIGURE 1:**
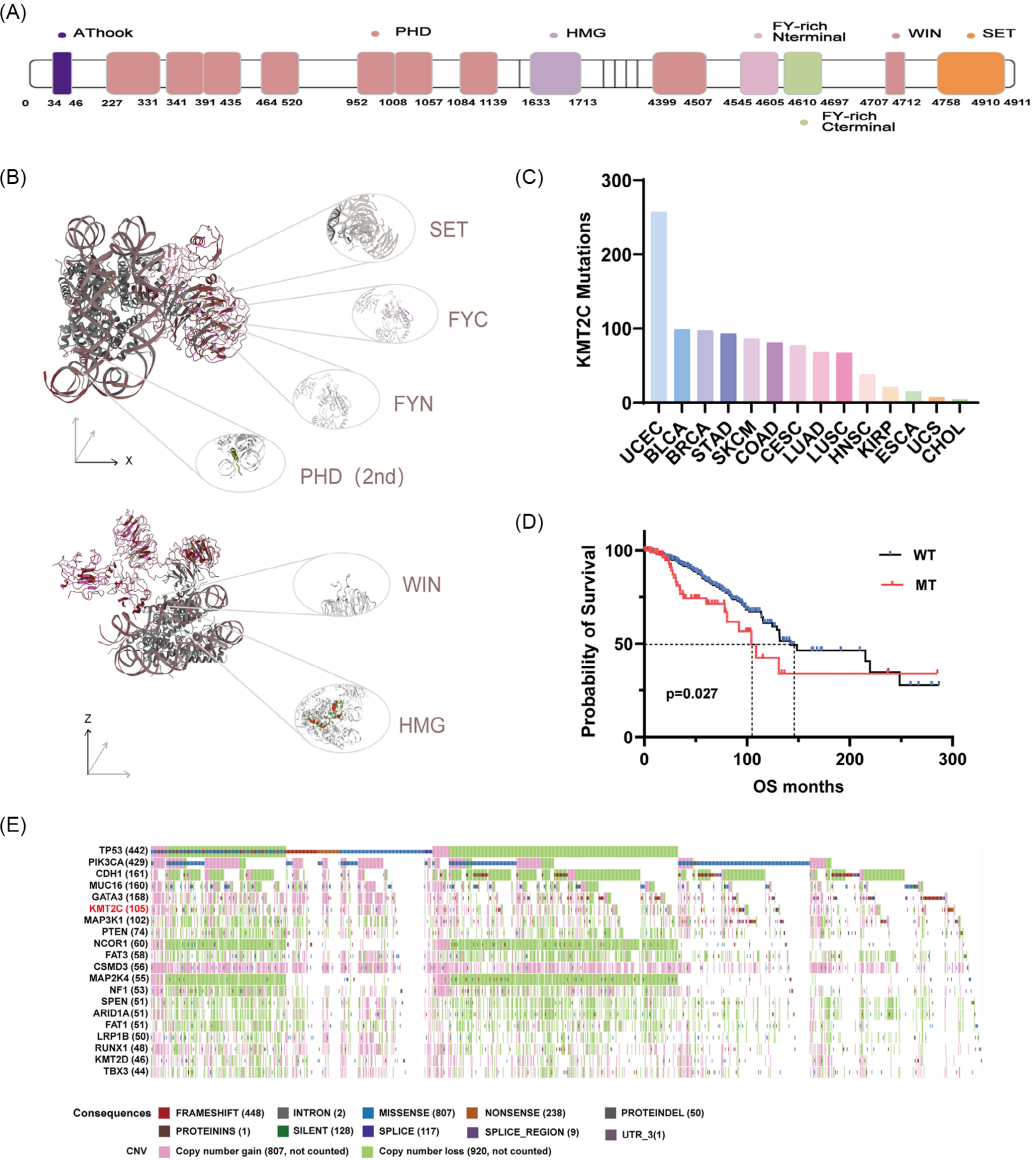
Analysis of the sequences, domains and mutation‐related situations of KMT2C. (A) Schematic diagram of the KMT2C domain in the linear sequence. (B) Residue fit view based on Q score of the 6kiw model diagram (Cryo‐EM structure of human MLL3‐ubNCP complex (4.0 angstrom)). (C) The cancer distribution of KMT2C mutations in 1083 cases affected. (UCEC: endometrial cancer; BLCA: bladder urothelial carcinoma; BRCA: invasive breast cancer; STAD: gastric adenocarcinoma; COAD: colon cancer; CESC: cervical squamous and adenocarcinoma; LUAD: lung adenocarcinoma; LUSC: lung squamous carcinoma; HNSC: head and neck squamous cell carcinoma; KIRP: renal papillary cell carcinoma; ESCA: esophageal cancer; USC: uterine sarcoma; CHOL: cholangiocarcinoma). (D) Kaplan–Meier survival analysis of TCGA‐BRCA cohorts, comparing patients with wild‐type (WT, *n* = 915) versus mutant (MT, *n* = 105) KMT2C. (E) OncoMatrix analysis of the 20 most frequently mutated genes (mutation counts bracketed) in the TCGA cohort (*n* = 1305). Mutation classes and copy number variations are indicated by the colour scales.

KMT2C (MLL3), located at chromosome 7q36,^16^, ^17^ encodes a 4911‐amino acid nuclear protein harbouring multiple functional domains, including an AT‐hook DNA‐binding motif, an HMG domain, FYRC/FYRN domains, several PHD‐type zinc fingers, and a catalytic SET domain^18^, ^19^, ^20^, ^21^ (Figure 1A and B). The C‐terminal SET domain serves as the core catalytic region, mediating the methylation of histone H3K4. By transferring the methyl group from S‐adenosyl‐L‐methionine to the target lysine residue, it activates the transcription process.^22^, ^23^ Adjacent to the SET domain, the FYRN and FYRC motifs contribute to maintaining the structural integrity and proper folding of the catalytic region, ensuring enzymatic stability and substrate specificity. In addition, multiple PHD‐type zinc finger domain functions as chromatin‐interacting modules, recognising specific histone features and facilitating the spatial confinement of KMT2C activity to enhancer regions enriched in H3K4me1.^24^, ^25^, ^26^ At the N‐terminus, the AT‐hook domain binds AT‐rich DNA,^27^ while the HMG domain stabilises the KMT2C/KMT2D complex by enhancing the nuclear retention of KDM6A (lysine‐specific demethylase 6A, also called UTX).^28^ Collectively, these structural elements define the enzymatic competence and chromatin specificity of KMT2C.

### 2.2 The biological functions of KMT2C

**FIGURE 2:**
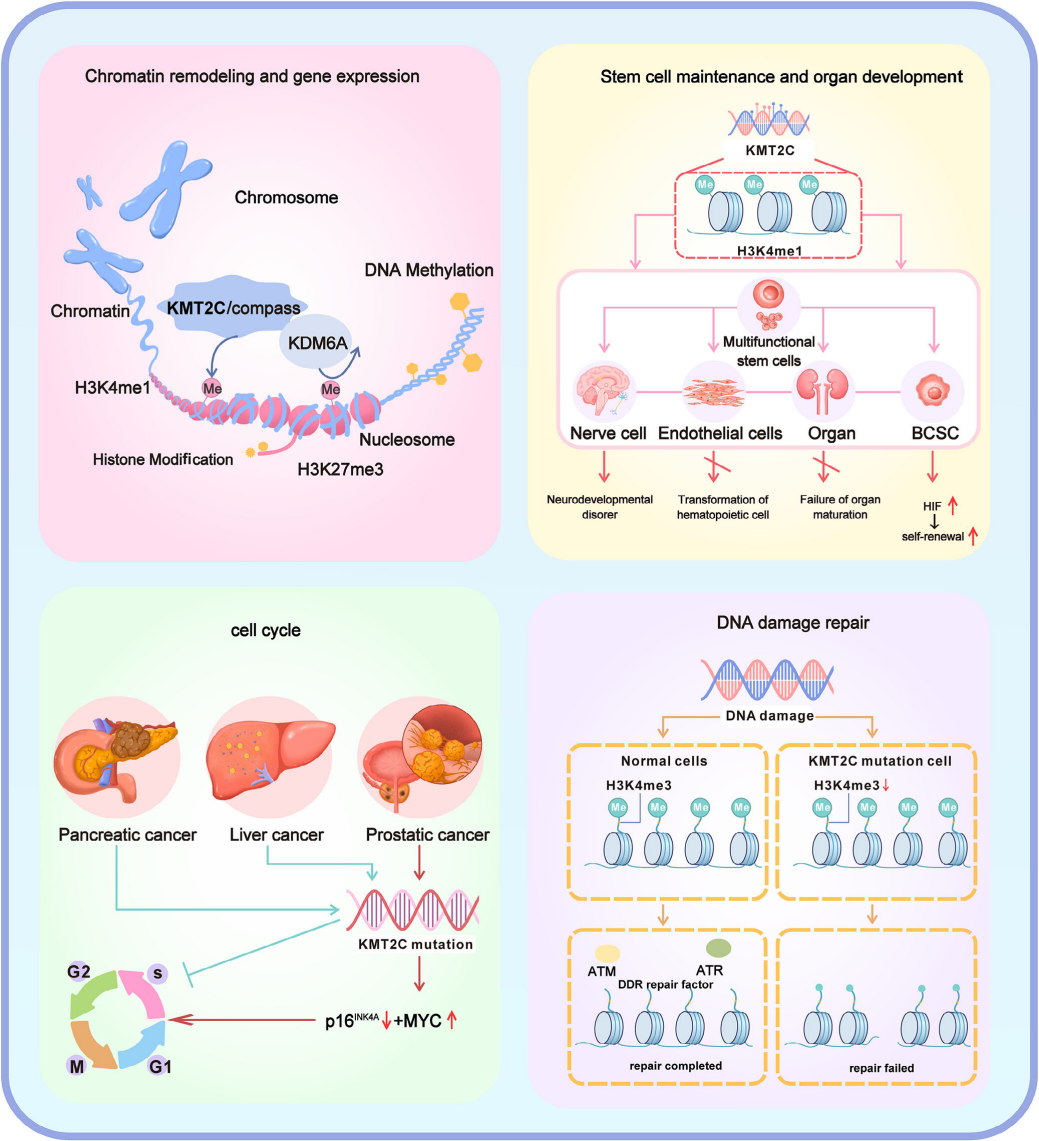
The biological functions of KMT2C. This figure systematically illustrates the central role of the histone methyltransferase KMT2C in maintaining cellular homeostasis and driving tumourigenesis. Under normal physiological conditions, KMT2C serves as the core catalytic component of the MLL3/COMPASS complex, depositing H3K4me1 marks to maintain chromatin accessibility and activate tumour suppressor gene expression. Simultaneously, it regulates stem cell differentiation equilibrium to ensure proper tissue development, while preserving genomic integrity through its involvement in DNA damage repair and cell cycle regulation. Following KMT2C mutation, functional loss leads to epigenetic imbalance (characterised by decreased H3K4me1 and increased H3K27me3), aberrant stem cell differentiation, compromised DNA repair mechanisms, and dysregulated cell cycle progression. These alterations collectively drive genomic instability and ultimately promote tumour initiation and progression.

KMT2C is a key epigenetic regulator that maintains cellular homeostasis by modulating chromatin accessibility and activating tumour suppressor and differentiation‐related genes. Its dysfunction contributes to tumour progression and invasion.^29^, ^30^, ^31^ KMT2C regulates tumour stem cell balance, mammary ducts morphogenesis, and epithelial cell differentiation through various pathways.^32^ It regulates cell proliferation, ensures genomic stability by homologous recombination repair. And its role in stem cell maintenance, differentiation, and DNA repair is environmentally dependent. In different types of cancer, it may exhibit either promoting proliferation or inhibiting growth effects.^33^, ^34^, ^35^ This section will systematically summarise the core regulatory roles of KMT2C in chromatin remodelling and gene expression, stem cell maintenance and organ development, cell cycle, and DNA damage repair (Figure 2).

#### 2.2.1 Chromatin remodelling and gene expression

The KMT2C gene mainly plays a key role in chromatin remodelling through its histone methyltransferase activity and its synergistic effect with other epigenetic regulatory factors. As a member of the COMPASS family, KMT2C mainly catalyses the monomethylation (H3K4me1) of histone H3K4, marking active enhancer regions.^21^, ^36^, ^37^, ^38^ These modifications destabilise nucleosomes, increase chromatin accessibility, and promote the assembly of transcriptional regulators and RNA polymerase II.^29^, ^30^, ^31^ Beyond its established role at enhancers, KMT2C also localises to promoter regions, where it modulates H3K4 trimethylation (H3K4me3) levels and contributes directly to transcriptional initiation, particularly for genes implicated in the maintenance of genome integrity and DNA repair processes.^39^

KMT2C regulates gene expression by promoting H3K4me1 methylation and activating H3K27 acetylation, while simultaneously removing the inhibitory histone mark H3K27me3.^40^ This process is mechanistically dependent on its synergy with KDM6A (UTX). KDM6A is a histone demethylase and it, along with KMT2C (MLL3), is a key component of the MLL3/4 complex, jointly participating in the regulation of enhancer activity. In the context of enhancers that are either inactive or pending activation, KDM6A, via its H3K27 demethylase activity, catalyses the removal of H3K27me3/2 marks. This demethylation is followed by the recruitment of the MLL3/4 complex, which deposits the H3K4me1 mark, thereby initiating the transition of the enhancer to an activated state.^41^, ^42^, ^43^ This activation is essential for promoting interactions between DNA and other genomic regions,^44^ a key process in the regulation of genes and cell division, cellular proliferation, differentiation, and organogenesis.

#### 2.2.2 Stem cell maintenance and organ development

The self‐renewing and highly proliferative nature of cancer stem cells is central to tumour growth, dissemination, and relapse. Research has demonstrated that KMT2C‐mediated histone H3K4 methylation modification serves a crucial function in the self‐renewal process of stem cells.^40^ In mammary tissue, KMT2C, as an epigenetic regulator, participates in the maintenance of stemness and lineage differentiation of mammary stem cells through the preservation of chromatin homeostasis. Functional deficiency of KMT2C leads to abnormal expansion of basal cells, impaired differentiation of luminal cells, and enhanced self‐renewal capacity of cancer stem cells via activation of the HIF signalling pathway, thereby driving tumourigenesis and progression.^32^ In pluripotent stem cells, the absence of KMT2C disrupts epigenetic stability, impairs the transformation of endothelial cells to haematopoietic cells,^45^ and differentiation of embryonic stem cells to extraembryonic cells.^46^ During neural development, KMT2C collaborates with KMT2D to catalyse the modification of H3K4me1, establishing an enhancer‐promoter network, and its absence delays the differentiation of neural precursor cells and affects the expression of neural‐related genes, ultimately affecting synaptic plasticity.^16^, ^47^, ^48^ In lung development, KMT2C regulates the differentiation of type II alveolar cells to type I, and its absence leads to perinatal death.^49^

#### 2.2.3 Cell cycle

The loss of KMT2C function results in markedly distinct cell cycle phenotypes across different cancers. This context‐dependent phenomenon stems from the complexity of its epigenetic regulatory function. Accumulating evidence indicates that the functional consequences of KMT2C dysregulation are strongly influenced by cell type–specific transcriptional programs and the underlying genetic background. In PTEN‐deficient prostate cancer, KMT2C mutations lead to epigenetic silencing of the p16^INK4A^ gene (within the Cdkn2a locus) and concurrently enhance MYC signalling activity. These combined effects promote cell cycle progression and overcome cellular senescence.^33^ However, in other contexts such as pancreatic ductal adenocarcinoma and liver cancer, where such synergistic genetic events are absent, KMT2C deficiency primarily alters the expression of a different set of key cell cycle regulators. This can lead to either the downregulation of cell cycle genes or a distinct cell cycle phenotype such as S‐phase arrest.^34^, ^35^ This tissue‐specificity demonstrates that the ultimate impact of KMT2C on the cell cycle is not intrinsic to the protein itself, but is shaped by the specific gene sets it regulates in different cellular contexts, as well as by its crosstalk with other signalling pathways. Despite these divergent mechanisms, KMT2C abnormalities consistently disrupt cell cycle homeostasis, underscoring its complex yet crucial role in tumourigenesis.

#### 2.2.4 DNA damage repair

Under normal physiological conditions, KMT2C facilitates DNA repair by recruiting DNA repair‐related proteins to the damage site^50^ and introducing H3K4 methylation to boost repair gene expression. In cancer cells, low KMT2C levels hinder DNA repair, increasing genomic instability.^51^ Theodoros Rampias discovered in bladder cancer research that KMT2C can directly bind to the promoters of key DNA damage response (DDR) and homologous recombination (HR) repair genes (such as ATM, ATR, BRCA1, and BRCA2), thereby regulating the transcription of these genes. When KMT2C is absent, the expression of these genes significantly decreases, and the methylation levels related to the promoters also decline. This epigenetic and transcriptional dysregulation ultimately leads to the inhibition of 1368 downstream genes, thereby induces genomic instability through defective DNA repair.^39^ Therefore, KMT2C is essential for preserving genome stability, and its reduced expression is closely linked to malignant cellular phenotypes.

### 2.3 Pathological roles of KMT2C loss

KMT2C regulates lineage‐specific epigenetic programs through H3K4 methylation, and genetic ablation studies in cells and animals have revealed its context‐dependent functions. In developmental contexts, KMT2C knockout in human pluripotent stem cell (hPSC) disrupts haemogenic endothelium differentiation via aberrant NODAL activation,^45^ while its variants are genetically linked to ovarian failure.^52^ Pathologically, KMT2C acts as a potent tumour suppressor; its ablation in urothelial cells promotes oncogenesis by disturbing enhancer‐mediated transcription.^53^ In cancer models, CRISPR‐mediated KMT2C loss enhances brain metastasis in triple‐negative breast cancer through a KDM6A–MMP3 axis^54^ and promotes androgen deprivation–induced luminal‐to‐double‐negative lineage transdifferentiation in prostate cancer by destabilising AR lineage maintenance and activating ΔNp63–HRAS/MAPK signalling.^55^ Together, evidence from cellular and in vivo studies highlights KMT2C as a key regulator of transcriptional homeostasis and a potential therapeutic target.

## 3 MUTATION AND DYSFUNCTION OF KMT2C IN BREAST CANCER

### 3.1 KMT2C mutations in breast cancer

KMT2C is a tumour suppressor frequently mutated in multiple solid tumours, with notable prevalence in breast cancer, endometrial cancer, and head and neck squamous cell carcinoma, among others^15^, ^56^, ^57^, ^58^, ^59^, ^60^, ^61^, ^62^, ^63^ (Figure 1C). These mutations mainly affect key functional regions, such as the SET and PHD regions, leading to impaired activity.^64^, ^65^, ^66^ As a result, KMT2C's ability to bind enhancer regions of tumour suppressor genes is impaired, causing epigenetic dysregulation and disrupting cellular homeostasis.^67^

In breast cancer, alterations in KMT2C—such as mutations, copy number loss, and decreased transcript levels—are linked to poor prognosis, aggressive subtypes, and endocrine therapy resistance.^68^, ^69^, ^70^ Overall, patients harbouring KMT2C mutations exhibit significantly shorter median survival than those with wild‐type tumours (104.2 vs. 142.2 months, Figure 1D and E). Consistent with this observation, ER‐positive patients with low KMT2C and high SLC20A1 expression demonstrate extremely poor long‐term outcomes, with a 10‐year survival rate of 0%, whereas high KMT2C and low SLC20A1 expression is associated with an estimated 86.78% survival at 10 years.^69^

Large‐scale cohort analyses further indicate that the prognostic impact of KMT2C mutation is context dependent. In the TCGA cohort, KMT2C mutations correlate with poorer outcomes in breast cancer overall (*p *= .027) and with reduced overall survival in the HR+ subtype (*p *= .016). In contrast, this association is not observed in the METABRIC cohort, either in the overall population or within HR+ tumours, underscoring substantial inter‐cohort heterogeneity.^66^

From a genomic perspective, KMT2C mutations are more frequent in HR+/HER2– subtypes, with a prevalence of up to 30.8% in invasive lobular breast cancer.^66^ Additionally, racial and ethnic differences have been observed; for example, the mutation rate in a Thailand TNBC cohort reached 58%, compared to 8% in the general TNBC population.^71^ KMT2C mutations often co‐occur with other driver alterations such as CDH1 and display subtype‐specific and clonal heterogeneity, highlighting their relevance in molecular subtyping and personalised treatment strategies.^72^ Beyond breast cancer, emerging evidence also implicates KMT2C dysfunction in the progression of other malignancies, including small cell lung cancer and colorectal cancer, further supporting its broad potential as a biomarker and therapeutic target across cancer types.^73^, ^74^

### 3.2 Molecular mechanism of KMT2C in breast cancer

#### 3.2.1 Epigenetic regulation

In breast cancer, KMT2C mutations primarily include frameshift, truncation and missense variants,^70^ which usually occur in exon regions and disrupt key functional domains, resulting in the loss of enzymatic activity.^75^ This affects H3K4 methylation and downregulates tumour suppressor gene expression. For instance, Lu Wang et al. demonstrated that knockdown of KMT2C in the human breast cancer cell line CAL51 led to the downregulation of several tumour suppressor genes, including GRHL2 and FRZB. Concurrently, KMT2C deficiency was shown to enhance cellular growth and colony formation in vitro. Furthermore, in vivo studies revealed that KMT2C loss significantly accelerated tumour growth and shortened overall survival in animal models.^26^

#### 3.2.2 Breast cancer stem cells and tumour heterogeneity

Breast cancer stem cells (BCSCs) initiate and drive tumour growth and spread by self‐renewal and differentiating into multiple cell types, contributing to tumour diversity and drug resistance.^76^, ^77^, ^78^ Studies have shown that KMT2C plays a crucial role in inhibiting the characteristics of breast cancer stem cells by maintaining the cell‐specific enhancer landscape. Its functional deficiency leads to extensive reprogramming of enhancers: in ERα+ breast cancer, the absence of KMT2C directly results in the inactivation of enhancers related to the ERα signalling pathway (such as the decrease in H3K4me1 and H3K27ac levels), forcing tumour cells to break away from estrogen dependence.^70^ Meanwhile, in a low‐oxygen microenvironment, the loss of KMT2C function can activate the hypoxia‐inducible factor HIF signalling pathway, thereby enhancing the adaptability and stem cell characteristics of BCSCs.^51^ Notably, KMT2C's role varies by subtype; In inflammatory breast cancer (IBC), its deficiency induces widespread epigenetic dysregulation and accelerates clonal evolution, particularly in HR+ subtypes, where it cooperates with PTEN and FBXW7 mutations to increase genetic diversity.^79^ Clinically, KMT2C mutations are associated with resistance to endocrine therapy in ER^+^ breast cancer, consistent with its dual role in modulating ERα enhancer activity and suppressing hormone‐independent transformation.^70^ Together, these findings elucidate the epigenetic mechanisms by which KMT2C regulates breast cancer stem cells (BCSCs) and heterogeneity, providing a molecular basis for precise treatment strategies targeting different subtypes.

#### 3.2.3 Tumour microenvironment

KMT2C plays a complex, context‐dependent role in shaping the tumour immune microenvironment, with effects varying by expression levels. In breast cancer, high KMT2C expression suppresses immunogenicity by inhibiting key immune activation pathways (TNF‐α, IFN‐γ, IFN‐α), fostering an immunosuppressive state.^80^ Conversely, KMT2C deficiency exhibits dual effects: it increases TMB and neoantigen production—enhancing cytotoxic T cell and macrophage responses and improving prognosis^31^, ^81^—but it also upregulates MMP3 and PD‐L1 through epigenetic regulatory mechanisms.^13^ Overall, the conflicting immune‐modulating effects represent a composite outcome of imbalanced biological regulation under different KMT2C expression states. Mechanistically, the absence of KMT2C will reduce the binding of RNA polymerase II to the PD‐L1 promoter, thereby affecting the transcriptional activity of PD‐L1, and simultaneously enhancing the sensitivity to PD‐L1 antibody therapy.^82^ However, single‐cell analyses reveal that KMT2C loss upregulates immune checkpoints (CD274), exacerbates T cell exhaustion, and disrupts Treg/Teff balance, thereby promoting tumour progression.^13^ These findings position KMT2C as a potential biomarker for immunotherapy response, with KMT2C‐deficient tumours possibly benefiting from combined checkpoint blockade and epigenetic therapy.

**FIGURE 3:**
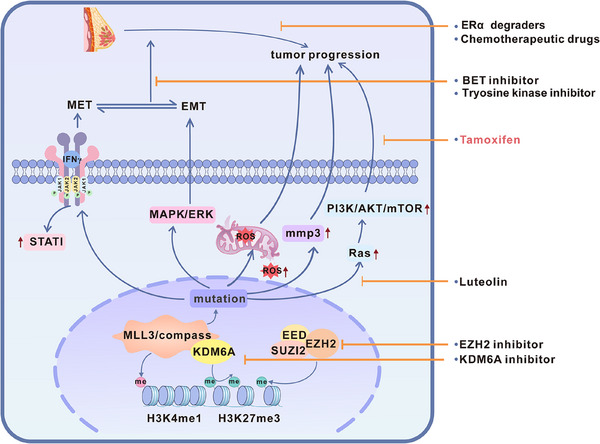
Molecular mechanisms by which KMT2C mutations promote breast cancer progression. (1) KMT2C mutations induce tamoxifen resistance by activating the PI3K/AKT/mTOR signalling pathway. The flavonoid luteolin upregulates KMT2C expression, restoring tumour cell sensitivity to tamoxifen. (2) KMT2C mutations activate the MAPK/ERK pathway, promoting a hybrid epithelial–mesenchymal transition (EMT) phenotype that accelerates tumour progression and enhances sensitivity to BET inhibitors and lapatinib. (3) KMT2C mutations upregulate IFNγ signalling, thereby enhancing MET pathway activity and facilitating distant metastatic colonisation. (4) KMT2C mutations impair mitochondrial function, increasing reactive oxygen species (ROS) levels and further driving tumour progression. (5) KMT2C mutations promote multi‐organ metastasis via the KDM6A–MMP3 axis, which can be reversed by the KDM6A inhibitor GSK‐J4. (6) KMT2C mutations induce global epigenetic imbalance, which can be partially restored by EZH2 inhibitors.

KMT2C acts as a central epigenetic regulator of breast cancer metastasis and drug response by modulating H3K4 methylation. It integrates IFNγ‐driven EMT/MET programs, crosstalk with major oncogenic pathways, and direct control of downstream effectors, thereby shaping metastatic behaviour and therapeutic sensitivity (Figure 3).

#### 3.2.4 IFNγ‐EMT/MET functional axis

Epithelial–mesenchymal transition (EMT) is a highly plastic biological program through which epithelial cells lose intercellular adhesion, polarity, and basement membrane attachment, acquiring mesenchymal phenotype such as cytoskeletal reorganisation, increased motility, and fibroblast‐like morphology under specific signal induction.^83^ EMT and its reverse process, MET, are central to tumour progression, enabling phenotypic plasticity that drives invasion, metastasis, and adaptation to the tumour microenvironment.^84^

Mutations in KMT2C and KMT2D are both related to upregulation of EMT‐related genes by altering the H3K4me1/3 landscape. In breast epithelial and lung cancer cell models such as HMLER, HCC827, and SUM149D2, the deletion of KMT2D disrupts the genomic localisation of PRC2 (including reduced binding to ZEB1/2 promoters), thereby relieving the inhibition of several EMT transcription factors. Cells lacking KMT2D enter a highly mesenchymal state (with increased expression of PRRX1 and complete loss of keratin), lose their cell plasticity and proliferation colonisation ability, and thus are not conducive to metastasis,^85^ while knockdown of KMT2C promotes a hybrid EMT‐MET phenotype that favours metastasis.^12^ Studies by Ban and KVOKAČKOVá further confirmed that EMT enhances invasion, stemness, and chemoresistance in breast cancer, contributing to tumour heterogeneity and treatment failure.^86^, ^87^ Mechanistically, loss of KMT2C during metastasis increases H3K27ac at enhancer of interferon‐γ (IFNγ) response genes, upregulating key mediators such as STAT1 and sensitising cells to EMT/MET signals. This promotes a hybrid EMT state and enhances metastasis colonisation. To identify potential molecular vulnerabilities associated with KMT2C deficiency, chemical screening studies comparing syngeneic wild‐type and KMT2C‐mutant cells revealed that KMT2C‐mutant cells are markedly more sensitive to the BET inhibitors,^12^ highlighting a potential therapeutic strategy for targeting KMT2C‐mutant breast cancers.

#### 3.2.5 MAPK/ERK pathway

Extensive studies have demonstrated that abnormal activation of the MAPK/ERK signalling pathway significantly promotes tumour progression and metastasis.^88^, ^89^, ^90^, ^91^ In breast cancer models, including MDA‐MB‐231 and MCF‐7, this pathway enhances migration and invasion.^92^ Mechanistically, MAPK/ERK activation initiates a cascade that drives uncontrolled proliferation, inhibits apoptosis, induces 5‐FU resistance, and promotes epithelial–mesenchymal transition (EMT), thereby accelerating malignant procession.^89^

However, some animal studies have revealed that KMT2C deletion in breast cancer reduces H3K4me1 and H3K4me3 levels, resulting in increased expression of EMT‐associated transcription factors, including Snai1, Snai2, and Zeb1. These factors not only facilitate extracellular matrix remodelling but also trigger mitochondrial dysfunction. Further mechanistic studies have shown that KMT2C deletion activates the MAPK/ERK signalling pathway, thereby accelerating tumour formation. Interestingly, KMT2C‐deficient cells exhibit increased sensitivity to lapatinib, highlighting the dual role of KMT2C in regulating oncogenic signalling and therapeutic response through the interaction between epigenetic remodelling and kinase pathways.^93^

#### 3.2.6 KDM6A–MMP3 functional axis

While KDM6A is widely recognised as a tumour suppressor in numerous cancers by removing the repressive H3K27me3 mark to activate tumour suppressor genes, its function is highly context‐dependent. Emerging evidence indicates that the role of KDM6A can undergo a fundamental switch between tumour‐suppressive and oncogenic functions, depending on the specific genetic background and tumour type. A striking example is provided by Guo et al., who demonstrated that in hepatocellular carcinoma (HCC), KDM6A acts as an oncogene by upregulating FGFR4 expression, triggering PI3K/AKT/mTOR signalling, and promoting tumour progression and metabolism reprogramming.^94^ This functional duality of KDM6A is also critically involved in breast cancer metastasis, particularly through the regulation of matrix metalloproteinase 3 (MMP3). Overexpression of MMP‐3 is significantly associated with poor tumour prognosis. It promotes invasion by degrading extracellular matrix components (e.g., collagen, fibronectin) and activating MMP‐1/9 in a cascade manner.^95^ MMP‐3 promotes metastasis by reshaping the tumour microenvironment, stimulating angiogenesis, and supporting immune evasion, ultimately leading to a high‐invasive clinical phenotype.^96^ Deletion of KMT2C leads to alterations in the levels of H3K4me1 and H3K27me3, while increasing H3K27ac, thereby forming an open chromatin structure. This enhances KDM6A binding and facilitates MMP3 transcription by removing repressive epigenetic marks and recruiting the acetyltransferase P300. Functionally, MMP3 promotes tumour cell brain metastasis by degrading the extracellular matrix and disrupting the blood–brain barrier. Clinical data show that in patients with KMT2C mutant TNBC, the expression level of MMP3 is significantly increased, and it is significantly positively correlated with the risk of brain metastasis. This finding is highly consistent with the previous experimental findings.^13^, ^97^

Notably, recent studies further reveal that KDM6A depletion in breast epithelial cells reduces sensitivity to anticancer agents while concomitantly activating TGFβ signalling, a central driver of epithelial–mesenchymal transition and therapy resistance.^98^ Collectively, these observations indicate that KDM6A can generate distinct—and potentially opposing—oncogenic outputs depending on the molecular and epigenetic context, underscoring the necessity for subtype‐ and pathway‐informed strategies when considering KDM6A‐targeted therapeutic interventions.

#### 3.2.7 RAS/PI3K/AKT/mTOR signalling pathway

Aberrant PI3K/AKT/mTOR signalling is a key driver of therapeutic resistance in breast cancer, particularly in HR^+^/HER2^−^ subtypes, frequently arising from PIK3CA mutations that promote tumour cell survival, proliferation, and endocrine resistance.^99^, ^100^ In parallel, activating RAS mutations induce conformational changes that lock RAS proteins in a constitutively GTP‐bound state, resulting in persistent PI3K/AKT/mTOR signalling, uncontrolled cell growth, and evasion of apoptosis.^101^ Emerging evidence indicates that KMT2C acts as an upstream epigenetic regulator restraining Ras‐driven signalling, and that its dysfunction contributes to endocrine resistance. Han‐Tsang Wu et al. confirmed the pivotal role of Ras‐driven PI3K/AKT/mTOR signalling in drug resistance and showed that restoration of KMT2C‐dependent enhancer activity can reverse tamoxifen resistance. In MCF7‐TamR cells, luteolin treatment increased KMT2C‐mediated H3K4me1 enrichment at the enhancer and promoter regions of RAS family genes, while concomitantly reducing Ras mRNA and protein expression. This Ras suppression attenuated PI3K/AKT/mTOR pathway phosphorylation and promoted apoptosis in breast cancer cells.^102^ Collectively, these findings suggest that loss or impairment of KMT2C disrupts enhancer‐mediated control of Ras signalling, leading to sustained PI3K/AKT/mTOR activation and the development of endocrine therapy resistance.

## 4 THERAPEUTIC PERSPECTIVES OF KMT2C IN BREAST CANCER

### 4.1 KMT2C as an innovative therapeutic target

KMT2C plays a crucial epigenetic role in inhibiting breast cancer metastasis. It inhibits cell migration by activating the TNS3 enhancer, while its loss reduces TNS3 expression and upregulates migration‐related genes such as ADAM12 and COL5A3, thereby enhancing metastasis.^103^ KMT2C mutations are common in breast cancer and correlate with elevated tumour mutational burden, reflecting greater genomic instability. Clinically, both somatic KMT2C alterations and reduced expression independently predict worse overall and disease‐free survival, underscoring KMT2C loss as an adverse prognostic factor.^81^ These findings not only reveal the key regulatory role of KMT2C in breast cancer metastasis but also provide important evidence for its potential as an innovative therapeutic target.

### 4.2 KMT2C and breast cancer treatment response

KMT2C plays a complex role in regulating endocrine therapy resistance in breast cancer, primarily through modulation of the estrogen receptor (ERα) signalling pathway. Mechanistically, KMT2C forms complexes with ERα and FOXA1 at enhancer of ERα target genes (such as c‐MYC and TFF1), affecting gene expression by regulating ERα binding.^30^ Additionally, its H3K4 methyltransferase activity facilitates active chromatin formation at the ERα promoter, upregulating ERα expression and maintaining the estrogen‐dependent growth. In tamoxifen‐resistant cells, elevated KMT2C expression enhances ERα‐dependent cell proliferation and attenuates treatment efficacy.^104^ As a COMPASS complex member, KMT2C also supports the TET2‐ERα positive feedback loop by promoting TET2 enhancer activity under estrogen stimulation. TET2, in turn, promotes ERα recruitment through DNA demethylation, which leads to the destruction of this loop and contributes to endocrine resistance.^105^ Notably, ER+ breast cancer cells with KMT2C deletion or mutation can still proliferate under estrogen deprivation conditions, showing a shortened resistance to aromatase inhibitors,^106^ and this growth may occur when no hormones are present, as ERα moves to AP‐1 genes. It is worth noting that although these cells acquire hormone‐independent growth ability, they still remain ERα‐dependent, so they are sensitive to selective estrogen receptor degraders (SERDs) such as fulvestrant,^107^ which provides important guidance for the clinical management of KMT2C‐mutation patients.

### 4.3 New therapeutic strategies for KMT2C mutant breast cancer

Therapeutic approaches for KMT2C‐mutant breast cancer emphasise multi‐target strategies. Although no KMT2C‐specific inhibitors are available, preclinical cell and animal studies indicate that several agents show therapeutic potential, offering new avenues for treatment. These findings provide important evidence for the combined application of chemotherapy drugs with epigenetic regulators, DNA damage response (DDR) inhibitors, and other targeted pathway inhibitors in treatment (Table 1).

**TABLE 1:** Therapeutic strategies for KMT2C mutant tumours.

Types of inhibitors	Inhibitor name	Methods	Main cell/animal model	Diseases	Effects after knockdown/knockout	Post‐administration effects	References
BET inhibitor	JQ1 GSK525762	CRISPR	MCF‐7 MDA‐MB‐231 NOD/SCID mice FVB/n mice C57BL/6 mice MaSC	BC	Enhanced tumour metastasis and invasion ability. Promote the formation of mixed epithelial–mesenchymal transition (EMT) state. Activate the IFNγ signalling pathway.	BET inhibition effectively suppressed the growth of KMT2C‐mutant primary tumours and metastases.	^12^
KDM6A inhibitor	GSK‐J4	CRISPR/Cas9	168FARN 67NR BALB/c mice NSG mice	BC	KMT2C‐mutant TNBC have higher MMP3 levels. Promoted multiple organ metastasis (especially brain metastasis). Alter the tumour immune microenvironment.	Reduce MMP3 levels. The brain metastasis is suppressed.	^13^
Chemotherapeutic drugs	Doxorubicin Cisplatin Carboplatin	siRNA	MCF‐7	BC	The cell proliferation ability declines. The gene expressions of DNA replication, homologous recombination and mismatch repair signalling pathways decrease.	Compared with the wild type, KMT2C knockdown in MCF‐7 cells increased their sensitivity to chemotherapy drugs and reduced cell survival rate.	^14^
HIF inhibitor	Acriflavine	CRISPR/Cas9 shRNA	Mammary stem cell (MaSC) organoid	BC	Activate the HIF pathway Enhance the activity of breast stem cells and impede their differentiation. Accelerate the occurrence of PI3K‐driven tumours.	Decline in the ability to form organoids.	^32^
PARP1/2 inhibitor	Olaparib	shRNA	HTB9 T24 NOD/SCID mice	BLCA	Influencing genes related to DNA damage response, repair, replication, cell cycle and apoptosis. DNA repair pathways depend on alterations.	Inhibit tumour growth and proliferation of tumour cells. Cause DNA damage and apoptosis.	^108^
Selective ERα degraders	Fulvestrant AZD9496 ARN1917 GDC927 RU58668	siRNA shRNA CRISPR/Cas9	MCF‐7 T47DCama‐1 Conditional KMT2C knockout mice	BC	Inhibition of proliferation. Loss of H3K4me1 and H3K27ac marks in the ERα enhancer region. Reduced expression of estrogen‐dependent genes.	Additional treatment with fulvestrant resulted in a synergistic reduction of ERα levels and the growth of the cells.	^70^, ^104^
EZH2 inhibitor	GSK126	CRISPR/Cas9 shRNA	MDA‐MB‐453 CAL51 KMT2C‐depleted cells in nude mice	BC	Influence protein interactions. Downregulate tumour suppressor genes. Promote tumour cell growth and colony formation ability. Shorten the lifespan of animals.	Restore gene expression Reduce H3K27me3 levels Tumour volume reduction and prolonged overall survival period.	^26^
Small‐molecule tyrosine kinase inhibitors(TKI)	Lapatinib	CRISPR/Cas9	Erbb2/Neu drive breast cancer mouse KMT2C KO mice	BC	The occurrence time of tumours was significantly advanced. The levels of H3K4me1 and H3K4me3 are decreased, which leads to epigenetic and transcriptional changes. Induction of epithelial–mesenchymal transition (EMT), mitochondrial dysfunction and elevated levels of reactive oxygen species (ROS). Imbalance of ERK/MAPK signalling pathway	Tumour growth was inhibited The Ki67 index decreased. The phosphorylation levels of ERK1/2 and Snai1 were significantly lowered.	^93^

#### 4.3.1 Epigenetic regulators

##### EZH2 inhibitors

Loss of KMT2C reduces KDM6A recruitment, elevates H3K27me3 levels, suppresses enhancer activity, and consequently disrupts downstream gene expression.^40^ EZH2 inhibitors counteract this effect by blocking PRC2 catalytic activity, reducing H3K27me3 accumulation, partially restoring enhancer function, and thereby inhibiting malignant phenotypes in KMT2C‐deficient tumour cells.^109^ The research by Lu Wang et al. revealed the key role of EZH2 inhibitors in regulating epigenetic balance. The mutation of KMT2C gene disrupts its binding with BAP1, resulting in abnormal accumulation of H3K27me3 at enhancers, silencing tumour suppressor genes, and further promoting tumour progression. EZH2 inhibitors (such as GSK126) can induce cell death by blocking H3K27me3 modification and restoring the expression of up to 80% of genes suppressed by KMT2C deficiency, thereby reshaping epigenetic balance and inhibiting tumour growth.^110^

##### KDM6A inhibitor

During breast cancer progression, KDM6A removes the repressive H3K27me3 mark, sustains CDH1 promoter activity, reactivates EMT‐related genes, maintains the epithelial phenotype, and promotes tumour cell colonisation at distant sites. Joseph H Taube et al. found that inhibiting the activity of KDM6A can prevent MET and suppress metastasis.^111^ In addition, Marco Seehawer et al. proved that the KDM6A inhibitor GSK‐J4 indirectly downregulates MMP3 and significantly reduces brain metastasis of KMT2C‐deficient breast cancer cells in vivo. GSK‐J4 showed good tolerability in mice, with no evident toxicity in major organs, including the liver and intestine. These findings indicate that KDM6A inhibitors are expected to be used to curb the progression of brain metastasis of KMT2C‐mutant TNBC.^54^

##### BET inhibitors

BET inhibitors disrupt super‐enhancer‐driven transcription by displacing BRD4 from chromatin through competitive binding to acetylated lysine. This impairs pathways related to tumour cell proliferation, invasion, and survival, showing promising therapeutic effects, especially in TNBC.^112^ Jihong Cui et al. demonstrated that the BET inhibitors JQ1 and GSK525762 selectively target KMT2C‐deficient breast cancer cells. Both agents suppressed the self‐renewal of KMT2C‐deficient organoids in vitro and inhibited primary tumour growth and metastatic proliferation in vivo, highlighting BET inhibition as a promising therapeutic strategy for KMT2C‐mutant breast cancer.^12^

#### 4.3.2 DNA damage response (DDR) inhibitors

Theodoros Rampias et al. found that the suppression of the KMT2C gene in tumours leads to a defect in homologous recombination repair (HR), forcing tumour cells to depend on PARP1/2 and alt‐EJ pathways for genomic stability. In this context, PARP inhibitors (e.g., Olaparib) induce synthetic lethality by blocking PARP enzyme activity, leading to the accumulation of DNA single‐strand breaks (SSB) that convert to lethal double‐strand breaks (DSB), which cannot be effectively repaired via alt‐EJ, ultimately resulting in cell apoptosis.^39^ Further studies revealed that the tumour suppressor SCRN2 stabilises KMT2C by inhibiting its CHIP‐mediated ubiquitination degradation via interaction with the BAG2 (a BAG family molecular chaperone regulator factor 2), thereby enhancing the pro‐apoptotic gene BMF expression and sensitising TNBC to PARP inhibitors.^113^ These results indicate that KMT2C plays a crucial regulatory role of DNA repair and a potential biomarker for molecular classification, therapeutic stratification, and targeted intervention in breast cancer.^114^

#### 4.3.3 PI3K/mTOR inhibitors

KMT2C mutations are relatively frequent in TNBC, especially in the metabolically active C2 subtype, where they often coexist with PIK3CA and KMT2D mutations. These tumours exhibit enhanced metabolic activity, reduced immune infiltration, and advanced pathological stages, forming an ‘immune‐cold’ phenotype associated with poor immunotherapy response. The C2 subtype also has characteristics of highly variable metabolic pathways, lack of immune features, and poor prognosis. Therapeutic strategies targeting PI3K signalling and angiogenesis may help inhibit tumour growth and improve outcomes in this subgroup.^115^

### 4.4 Translational potential of KMT2C in breast cancer precision therapy

As an important epigenetic regulator, KMT2C holds great promise in the precision treatment of breast cancer. Advances in detection technologies, particularly high‐throughput sequencing methods such as whole exome sequencing and next‐generation sequencing, have enabled efficient identification of KMT2C mutations.^116^ To further explore its biological function, RNA sequencing technology and immunohistochemical analysis are commonly used to assess gene expression and protein levels.^103^, ^117^ Genomic profiling of KMT2C has potential guiding value for the molecular typing of TNBC, especially in the Thai population. Its frequent mutations suggest the role of epigenetic regulation and provide the development of new therapies targeting chromatin remodelling.^71^ However, the specific molecular mechanism by which KMT2C mutations affect treatment sensitivity remain unclear and require further investigation. In 2022, researchers developed a 12‐gene algorithm incorporating KMT2C mutational status to distinguish chemotherapy responders from non‐responders, especially in TNBC. This tool improves treatment decision‐making and reduces unnecessary chemotherapy.^10^ In parallel, liquid biopsy technology—such as circulating tumour DNA and circulating tumour cells—enable dynamic monitoring of KMT2C mutations, providing real‐time guidance for treatment adjustment and response evaluation.^118^ Although KMT2C has demonstrated considerable potential in precision therapy for breast cancer, targeted therapy for this gene is still in the exploration stage, and its clinical efficacy and safety need to be verified through larger‐scale clinical trials.

Future research should focus on the following directions: firstly, multi‐centre clinical studies are needed to clarify the clinical applicability of KMT2C mutations as diagnostic markers and prognostic indicators; secondly, the molecular regulatory network of KMT2C involved in tumour occurrence and development should be deeply analysed to provide theoretical basis for the development of new targeted drugs; and finally, multi‐omics data such as genomics, transcriptomics, and proteomics should be integrated to establish more precise individualised treatment prediction models. We believe that continued investigation of KMT2C will further uncover its potential in precision treatment strategies for breast cancer. By establishing a precise diagnosis and treatment system based on KMT2C mutation status, not only can the selection of existing treatment plans be optimised, but it may also provide important clues for the development of new targeted treatment strategies, ultimately achieving the clinical goals of improving treatment effects and improving patient prognosis.

## 5 SUMMARY

Breast cancer is a global health issue with increasing incidence and stagnant survival rates, primarily due to tumour metastasis. Epigenetics dysregulation, particularly involving the histone methyltransferase KMT2C, plays a crucial role in breast cancer development. KMT2C, part of the COMPASS complex, influences chromatin dynamics and gene expression through H3K4 monomethylation and trimethylation, affecting processes such as stem cell maintenance and cell fate determination. Mutations in KMT2C are linked to greater tumour heterogeneity, metastasis, and poor prognosis, providing insights into breast cancer progression.

KMT2C exhibits complex duality in breast cancer. It functions as a dual‐faced epigenetic regulator: it can promote tumour progression by repressing tumour‐suppressive programs and activating oncogenic circuits like the IFNγ‐EMT/MET functional axis, the MAPK/ERK and RAS/PI3K/AKT/mTOR signalling pathways, and the KDM6A–MMP3 axis. On the other hand, KMT2C loss may sensitise specific breast cancer subtypes to therapeutic agents, such as EZH2 inhibitors, KDM6A inhibitors, and BET inhibitors. This functional duality makes it a current research hotspot.

However, the complex regulatory network and pathway cross‐talk involving KMT2C pose significant challenges for drug development. Several critical scientific questions remain to be elucidated, including whether the aforementioned functional axes exhibit synergistic interactions that collectively drive more potent metastatic programs. The multidimensional impact of KMT2C deficiency necessitates a strategic transition from monotherapy toward rational combination regimens—such as epigenetic inhibitors combined with chemotherapeutic agents—which require systematic validation in physiologically relevant models including patient‐derived organoids. Moreover, combining KMT2C‐related transcriptomic profiles with proteomic data can improve prognostic accuracy and guide more effective treatment decisions. Future research should focus on the following: (1) developing small‐molecule drugs that directly target KMT2C, with a focus on two strategic classes: protein stabilisers to prevent its degradation and functional surrogate compounds that can compensate for its loss by restoring its tumour‐suppressive transcriptional program; (2) exploring the therapeutic applications of gene editing technologies such as CRISPR/Cas9, while concurrently developing novel strategies to overcome current technical limitations, including inefficient and tumour‐specific in vivo delivery, potential off‐target genomic alterations, and unresolved safety concerns that currently constrain clinical translation; and (3) optimising the combined treatment regimens of traditional drugs and epigenetic regulators or other pathway regulators. These directions will promote the progress of precise treatment for breast cancer.

This review has comprehensively elucidated the molecular regulatory network of KMT2C, providing a foundation for developing novel targeted strategies in approaches for KMT2C‐mutant breast cancer. Future work should focus on validating these findings through in‐depth mechanistic research and well‐designed clinical trials to enable their translation into meaningful clinical benefits and improved patient outcomes.

## AUTHOR CONTRIBUTIONS

**Shuangmei Tong**: Writing—original draft. **Qiu Wang**: Writing—original draft. **Yun Chen**: Visualisation, Writing—review and editing. **Zunjie Bo**: Visualisation, Writing—review and editing. **Ya Zhang**: Writing—review and editing. **Yan Liu**: Writing—review and editing, Supervision, Conceptualisation, Project administration. Yan Liu supervised the study and provided overall direction during the manuscript preparation. Shuangmei Tong and Qiu Wang drafted and edited the manuscript. Yun Chen and Zunjie Bo designed the figures and tables. Yun Chen, Zunjie Bo, and Ya Zhang critically reviewed and revised the manuscript. All authors read and approved the final manuscript.

## ETHICS STATEMENT

Not applicable

## CONFLICT OF INTEREST STATEMENT

The authors declare no conflicts of interest.

## Data Availability

Not applicable.
